# Sequential PET/CT with [^18^F]-FDG Predicts Pathological Tumor Response to Preoperative Short Course Radiotherapy with Delayed Surgery in Patients with Locally Advanced Rectal Cancer Using Logistic Regression Analysis

**DOI:** 10.1371/journal.pone.0169462

**Published:** 2017-01-06

**Authors:** Biagio Pecori, Secondo Lastoria, Corradina Caracò, Marco Celentani, Fabiana Tatangelo, Antonio Avallone, Daniela Rega, Giampaolo De Palma, Maria Mormile, Alfredo Budillon, Paolo Muto, Francesco Bianco, Luigi Aloj, Antonella Petrillo, Paolo Delrio

**Affiliations:** 1 Radiation Oncology Unit, Istituto Nazionale Tumori “Fondazione G. Pascale” IRCCS, Napoli, Italy; 2 Nuclear Medicine Unit, Istituto Nazionale Tumori “Fondazione G. Pascale” IRCCS, Napoli, Italy; 3 Department of Economics, Universidad Carlos III, Madrid, Spain; 4 Pathology Unit, Istituto Nazionale Tumori “Fondazione G. Pascale” IRCCS, Napoli, Italy; 5 Gastrointestinal Medical Oncology Unit, Istituto Nazionale Tumori “Fondazione G. Pascale” IRCCS, Napoli, Italy; 6 Gastrointestinal Surgery Unit, Istituto Nazionale Tumori “Fondazione G. Pascale” IRCCS, Napoli, Italy; 7 Medical Physics Unit, Istituto Nazionale Tumori “Fondazione G. Pascale” IRCCS, Napoli, Italy; 8 Experimental Pharmacology Unit, Istituto Nazionale Tumori “Fondazione G. Pascale” IRCCS, Napoli, Italy; 9 Diagnostic Radiology Unit, Istituto Nazionale Tumori “Fondazione G. Pascale” IRCCS, Napoli, Italy; Universita Campus Bio-Medico di Roma, ITALY

## Abstract

Previous studies indicate that FDG PET/CT may predict pathological response in patients undergoing neoadjuvant chemo-radiotherapy for locally advanced rectal cancer (LARC). Aim of the current study is evaluate if pathological response can be similarly predicted in LARC patients after short course radiation therapy alone. Methods: Thirty-three patients with cT2-3, N0-2, M0 rectal adenocarcinoma treated with hypo fractionated short course neoadjuvant RT (5x5 Gy) with delayed surgery (SCRTDS) were prospectively studied. All patients underwent 3 PET/CT studies at baseline, 10 days from RT end (early), and 53 days from RT end (delayed). Maximal standardized uptake value (SUVmax), mean standardized uptake value (SUVmean) and total lesion glycolysis (TLG) of the primary tumor were measured and recorded at each PET/CT study. We use logistic regression analysis to aggregate different measures of metabolic response to predict the pathological response in the course of SCRTDS. Results: We provide straightforward formulas to classify response and estimate the probability of being a major responder (TRG1-2) or a complete responder (TRG1) for each individual. The formulas are based on the level of TLG at the early PET and on the overall proportional reduction of TLG between baseline and delayed PET studies. Conclusions: This study demonstrates that in the course of SCRTDS it is possible to estimate the probabilities of pathological tumor responses on the basis of PET/CT with FDG. Our formulas make it possible to assess the risks associated to LARC borne by a patient in the course of SCRTDS. These risk assessments can be balanced against other health risks associated with further treatments and can therefore be used to make informed therapy adjustments during SCRTDS.

## Introduction

In patients with LARC, SCRTDS is known to be a valuable therapeutic option. As compared to traditional neoadjuvant radiochemotherapy (NRC), SCRTDS leads to similar results in terms of the rate of R0 resection and satisfactory results in terms of downstaging and pathological response [1–3]. When compared to short course radiation therapy (SCRT), SCRTDS is known to lead to downsizing of the lesions ensuring a significant rate of pathological response [4–6] and can be considered in patients with locally advanced tumors unfit for chemo-radiation [7–9]. Unfortunately, not all patients benefit equally from neoadjuvant treatments and using new imaging modalities to make individual assessments of response to therapy could be of great clinical value to adjust subsequent strategies for each individual patient. Such strategies range from a tailored surgical approach, to administering an adjuvant regimen, or even to a wait and see policy without surgery for patients with high surgical risks [10, 11]. Conventional imaging modalities such as computed tomography (CT), magnetic resonance imaging (MRI), and endorectal ultrasound (EUS), successfully used for the initial staging of rectal cancer, perform poorly after neoadjuvant therapies, given that they are unable to accurately distinguish desmoplastic reactions or fibrosis from still viable tumors [12–14].

PET/CT with [^18^F]-FDG has been shown to predict response during NRC in LARC as well as in advanced esophageal cancer patients [15–19]. Nevertheless, few studies have addressed the evaluation of response with PET/CT with [^18^F]-FDG after SCRT [20–22] and to our knowledge no studies have evaluated the role of PET/CT results/parameters in predicting pathological response in the course of SCRTDS.

The aim of the current study is to investigate whether multiple semi quantitative parameters obtained from sequential PET/CT studies can be employed to assess the effects of preoperative radiation therapy using histopathology response as a gold standard for pathological response.

## Materials and Methods

### Patient characteristics

Thirty-three consecutive patients with histologically proven LARC, who refused or were considered unfit for chemo radiation and planned for treatment with neoadjuvant SCRTDS, were prospectively evaluated in this study. Patient characteristics are described in Table 1. Patients had T2-T3 rectal cancer with or without local lymph node involvement. Patients staged T2 without lymph node involvement were included only if the tumor was located at less than 5 cm from the anal verge.

**Table 1:** Patient descriptive characteristics.

Characteristics	Statistics	
**Sex**	No. of male	26
	No. of female	7
**Age**	Mean ± SD	68.8 ± 10.7
	Median	70
**cTNM** (clinical TNM)	T3N2	9%
	T3N1	46%
	T3N0	39%
	T2N1	3%
	T2N0	3%
**cCRM** (mm) (clinical Cinconferential Resection Margin)	n.d.	37%
	> 5	39%
	< 5	24%
**GR** (Gunderson Risk classification)	High	12%
	Moderately high	39%
	Intermediate	46%
	Low	3%
**Tumor Location** (cm from anal verge)	> 5	70%
	= 5	6%
	< 5	24%

Staging included EUS and/or MRI of the pelvis (with endorectal contrast media); contrast enhanced MRI of the liver, CT of the abdomen and pelvis and whole body PET/CT with [^18^F]-FDG. According to Gunderson’s risk of recurrence stratification [23], 15 patients were at intermediate risk, 13 at moderately high risk, 4 at high risk and only 1 at low risk.

Patients were included in the study in accordance with the approved guidelines of our ethical committee and gave their written informed consent.

### Preoperative radiotherapy

All patients underwent dose-planning CT in the prone position. CT images from the baseline PET/CT studies were used for treatment planning. The planning target volume (PTV) was generated according to ICRU recommendations [24, 25]. Three-dimensional plans were generated for a dual-energy (6 and 20MV x-rays) linear accelerator (Clinac 2100, Varian Medical Systems, Palo Alto, CA) equipped with multileaf collimators (MLC). Patients were planned using a 3 field arrangement to include the PTV within the 95% isodose and a dose of 25 Gy in 5 fractions over 1 week was prescribed to the ICRU 62 intersection point.

### PET/CT with [^18^F]-FDG

FDG PET/CT studies were acquired with a Discovery 600 hybrid scanner (GE Healthcare, Milwaukee, WI, USA) under standard fasting conditions with measured blood glucose levels below 150 mg/dl. No oral or intravenous contrast media were administered for the CT study.

**Fig 1:**
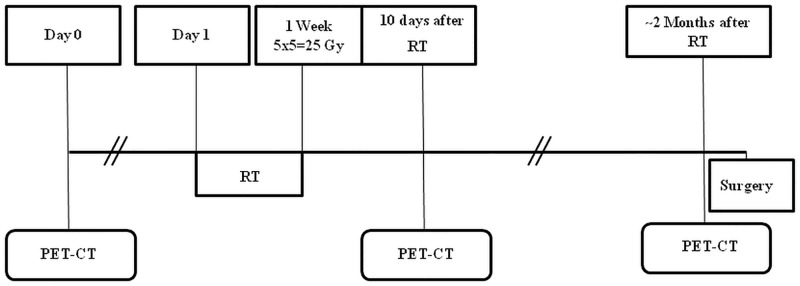
Schematic diagram of the Timing of PET/CT evaluation relative to treatment procedures.

**Fig 2:**
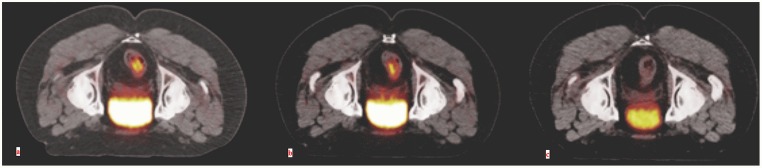
Sequential PET/CT studies in patient n. 2. Transaxial fused PET/CT images obtained in the prone position in the basal (a), early (b) and delayed (c) studies. A clear progressive reduction in FDG accumulation of the rectal lesion can be appreciated. This patient had a complete pathological response on pathological analysis (TRG1).

Each patient underwent 3 PET/CT studies: baseline, on average 10.7 days before starting radiotherapy; early, on average 10.1 days after the end of radiotherapy and delayed, on average 53 days after the end of RT and 7.3 days before surgery (Fig 1). The PET scan timings were chosen on the basis of previous published data on patients that underwent radiochemotherapy [15–17]. Volumes of interest (VOI) were drawn to define the extent of LARC and the relative analysis was performed using Volume Viewer software on a dedicated workstation (GE Advantage Workstation 4.4) by two experienced nuclear medicine physicians (Fig 2). The VOI was defined with a threshold method in which all pixels above a SUV value of 3 were included. The following parameters were recorded: SUVmax, defined as the maximum SUV value within the target volume; SUVmean, the average SUV value of all pixels included in the target volume; Metabolic Tumor Volume (MTV), the volume of all pixels in the target volume; Total Lesion Glycolysis (TLG), defined as the product of SUVmean and MTV.

### Surgery

Surgery was performed on average 60.3 days after the end of radiotherapy. Based on the results of restaging and downsizing, sphincter-saving surgery was considered for all patients without a clear sphincter involvement before treatment and local excision was considered for patients with a significant clinical response. The planned operation was discussed with the patients and a specific informed consent was obtained. A rectal resection with total meso-rectal excision and bilateral nerve sparing, when possible, was the standard approach. In distal cancers an ultra-low anterior resection with colo-anal manual anastomosis or, in case of sphincter involvement, an abdomino-perineal resection were performed. All patients receiving an anastomosis underwent construction of a protecting ileostomy.

### Pathology

Postsurgical pathology examination provided a macroscopic description of the mesorectum and of the former tumor-bearing area; at least four paraffin blocks were processed and an additional larger area block was embedded. If no tumor was visible, the entire suspicious area was sliced and embedded.

Tumor regression grade (TRG) was scored based on a five-point system, as previously reported, after being independently evaluated by two pathologists [26]. Lesions were scored from TRG 1 (complete pathologic response) to TRG 5 (clear signs of tumor progression). In case of discrepancy between the two pathologists, the worse TRG score was assigned. Patients were classified as pathological major responders (TRG1-2), complete responders (TRG1) or non-responders (TRG3-5) based on these findings.

### Statistics

The principal objective of this work is to predict the pathological response to SCRT on the basis of PET/CT parameters. We consider logistic regression models to estimate the probability of a major response (TRG1-2) or of a complete response (TRG1).

As compared to performing discrimination analysis, such as ROC analysis, this approach has two advantages:

- It makes it possible to classify response on the basis of *multiple* measurements rather than a single one;
- It provides a direct *estimate of the individual probability* of pathological response rather than simply discriminating between different histopathological responses.

To assess our results we address the properties of our model in terms of discrimination (how well predicted probabilities separate patients with different responses) and prediction accuracy (how well predicted probabilities agree with individual responses):

- Discrimination: We construct a Receiver Operating Characteristic (ROC) curve using the predicted probability of response for each of the patients. This is useful to clarify the ability to discriminate between responders and nonresponders.
- Prediction accuracy:
  - To assess the ability to make in-sample predictions we plot predicted probabilities of histopathological response against observed histopathological response and we report the (McFadden) Pseudo *R*^2^, a measure of the improvement in prediction ability above a model with only a constant term.
  - To assess the ability to make out-of-sample predictions, we perform leave-one-out-cross-validation (LOOCV), a commonly used validation method [27, 28]. This amounts to repeating the logistic regression 33 times. Each time one patient is left out for validation and the set of observations used to make a prediction is composed of the remaining 32 patients. This gives a better idea of the ability to predict pathological response on the basis of TLG measurements, because the probability of pathological response of each patient is computed without using their data.

All statistical analyses were performed using STATA/SE 11.2 software (Stata Corp. LP, College Station, TX, USA).

## Results

In the following we begin by giving a description of TLG measurements and their association with histopathological response. We then present the models to predict major response and complete response.

### TLG measurements

The comparisons between TLG measurements at different times were performed using the Wilcoxon signed-rank test. The average early reduction in TLG (reduction from baseline to early measurement) was 50% (*P* < 0, 0000). The average delayed reduction in TLG (reduction from early to delayed measurement) was 50% (*P* < 0, 0000). The average overall reduction in TLG (reduction from baseline to delayed measurement) was 83% (*P* < 0, 0000).

### TLG measurements and histopathological responses

Absolute values of TLG parameter levels and their changes after treatment were correlated with pathological response using the Kruskal-Wallis equality-of-populations rank test.

The TRG groups are statistically related to early and delayed measurement of TLG (*P* = 0.0001 and *P* = 0.0005, respectively), but not to baseline values (*P* = 0.1110). The TRG groups are also statistically related to early and overall reductions in TLG (*P* = 0.0119 and *P* = 0.0110, respectively), but not to delayed reductions (*P* = 0.6170).

Major response is statistically related to early and overall reductions in TLG. Average early reduction in TLG is 56% for responding tumors compared to 43% for nonresponding tumors (*P* = 0.0089). Average overall reduction in TLG is 88% for responding tumors, compared to 77% for nonresponding tumors (*P* = 0.0042).

Major response is not statistically related to delayed reductions in TLG. Average delayed reduction in TLG is 44% for responding tumors compared to 54% for nonresponding tumors (*P* = 0.8005).

### Predicting response

Our analysis indicates significant metabolic responses to SCRTDS (SUVmax, SUVmean, or TLG) already at the early PET and a clear correlation between these measurements of metabolic response and TRG. Nevertheless, in line with the findings in [20], reductions in individual SUV and TLG values are insufficient for a clinically useful classification of patients as major responders (TRG1-2) or nonresponders (TRG3-4):

- Reductions from baseline to early measurement classify correctly no more than 25 of 33 patients (75.76% of the total);
- Reductions from baseline to delayed measurement classify correctly no more than 27 of 33 patients, (81.82% of the total).

In contrast to these discouraging results, we find that a simple logistic regression model based on two measurements (the early measurement of TLG and the overall proportional reduction in TLG from the early to the delayed measurement) has a remarkable ability to classify patients and predict their histopathological response.

### Predicting major response

To predict major response we find that the best specification of a logistic model includes only the level of TLG at the early PET and the overall proportional reduction of TLG from the baseline to the delayed PET. We denote by *TLGBaseline*, *TLGEarly* and *TLGDelayed* the baseline, early, and delayed measurements of TLG and by *OverallReductionTLG* the proportional overall reduction in TLG between the baseline and the delayed measurement

$$OverallReductionTLG=\frac{TLGBaseline-TLGDelayed}{TLGBaseline}$$

Our results for the probability of major response (TRG1-2) computed with a logistic regression with *TLGEarly*, *OverallReductionTLG*, and a constant term are summarized in Table 2.

**Table 2:** Prediction of major response: Results of logistic regression analysis.

	Coefficient	Robust Std. Err.	*z*	*P* > |z|	95% Conf. Interval
*TLGEarly*	−.3198	.1215	−2.63	0.008	[−.55788, −.08162]
*OverallReductionTLG*	37.8934	14.3964	2.63	0.008	[9.6770, 66.1097]
*Constant*	−13.4911	5.0289	−2.68	0.007	[−23.3476, −3.6345]

The early measurement of TLG and the proportional overall reduction in TLG measurements have the expected signs (higher early measurement of TLG and smaller overall reductions in TLG measurements lead to a lower probability of pathological response) and are both significant. The constant term is also significant. Pseudo *R*^2^ is 0.8414.

On the basis of these results the probability of major response can be computed in the following way. First, use the coefficients in Table 2 to compute *X*, a linear combination of *TLGEarly* and *OverallReductionTLG* as in Eq (1)

$$\begin{array}{c} X=-13.4911-.3198*(TLGEarly)+37.8934*(OverallReductionTLG). \end{array}$$ (1)

Then, use *X* to compute the probability of response according to the following formula:

$$\begin{array}{c} \frac{1}{1+e^{-X}}. \end{array}$$

**Fig 3:**
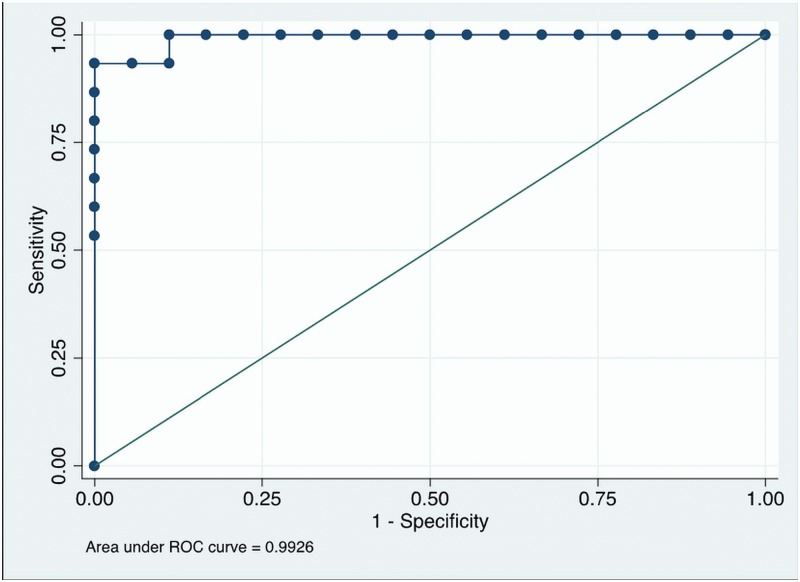
ROC Analysis for prediction of major response (TRG1-2).

We construct a ROC curve using the predicted probability of response for each of the 33 patients (Fig 3). This is useful to clarify the ability of the predicted probability of response to discriminate between major responders (TRG1-2) and nonresponders (TRG3-4). The result is shown in Fig 3. The area under the curve (AUC) is 0.9926 and its 95% confidence interval is [0.9750, 1.00000]. Setting a cut point of 0.5806 leads to 32 of the 33 patients (96.97%) being correctly classified (only one responding patient is incorrectly classified as nonresponding) and to sensitivity of 93.33% and specificity of 100%.

**Fig 4:**
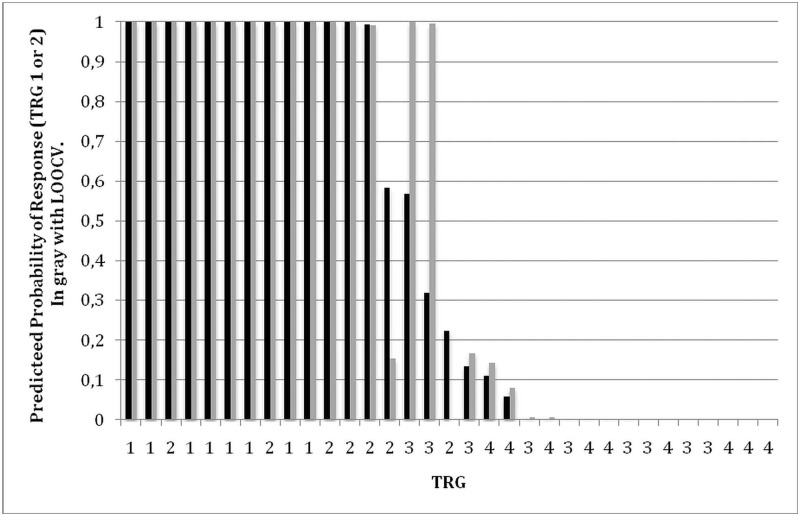
Prediction of major response. In sample (black bars, TRG1-2) and out of sample (LOOCV, gray bars, TRG1-2).

To assess in-sample prediction accuracy, for each individual patient we plot in Fig 4 the predicted probability of response (black bar) and their observed histopathological response. For 26 of 33 patients the model predicts the histopathological response with near certainty. For 13 of the 15 major responders (TRG1-2) the model predicts a probability of being a responder no lower than 0.9933 and for 13 of 18 nonresponders the model predicts a probability of being responder no higher than 0.004. For the remaining two responding patients (both with TRG2) the model predicts probabilities of being a major responder of 0.58 and 0.22 and for the remaining 5 nonresponders the model predicts probabilities of being a responder between 0.05 and 0.56.

To assess the ability of our model to make out-of-sample predictions, we performed leave-one-out-cross-validation (LOOCV). The results are reported in Fig 4 (gray bars). Cross validation leads to no notable changes when predictions are nearly certain (predicted probabilities very near 0 or 1). But LOOCV leads to different predictions in the case of 4 of the 7 patients for whom predictions were not nearly certain.

The previous analysis reveals that for most of the patients our model makes it possible to make predictions that are virtually certain and that are robust in the sense that the prediction is unchanged when the patient’s observation is dropped.

## Predicting complete response

To predict complete response we find that the best specification of a logistic model includes only the level of TLG at the early PET and the overall proportional reduction of TLG from the baseline to the delayed PET. Our results for the probability of complete response (TRG1) computed with a logistic regression with *TLGEarly*, *OverallReductionTLG* and a constant term are summarized in Table 3.

**Table 3:** Prediction of complete response: Results of logistic regression analysis.

	Coefficient	Robust Std. Err.	*z*	*P* > |z|	95% Conf. Interval
*TLGEarly*	−.0783	.0311	−2.52	0.012	[−.1393, −.01741]
*OverallReductionTLG*	24.9975	10.2491	2.44	0.015	[4.9097, 45.0853]
*Constant*	−21.5914	9.6301	−2.24	0.025	[−40.4660, −2.7168]

The early measurement of TLG and the overall reduction in TLG measurements have the expected signs (higher early measurements of TLG and smaller overall reductions in TLG measurements lead to a lower probability of a complete pathological response) and are both significant. The constant term is also significant. Pseudo *R*^2^ is 0.6710.

On the basis of these results the probability of complete response can be computed in the following way. First, use the coefficients in Table 3 to compute *Y*, a linear combination of *TLGEarly* and *OverallReductionTLG* as in Eq (2)

$$\begin{array}{c} Y=-21.5914-.0783*(TLGEarly)+24.9975*(OverallReductionTLG). \end{array}$$ (2)

Then, use *Y* to compute the probability of response according to the following formula:

$$\begin{array}{c} \frac{1}{1+e^{-Y}}. \end{array}$$

**Fig 5:**
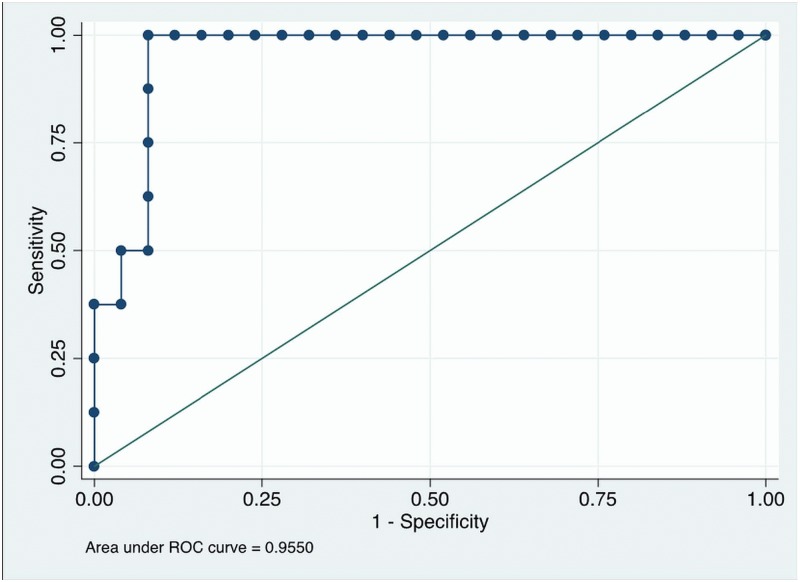
ROC Analysis for prediction of complete response (TRG1).

We construct a ROC curve using the predicted probability of complete response for each of the 33 patients. This is useful to clarify the ability of the predicted probability of response to discriminate between complete responders (TRG1) and not complete responders (TRG2-4). The result is shown in Fig 5.

The area under the curve (AUC) is 0.9550 and its 95% confidence interval is [0.8877, 1]. Using a cut point of .5580 gives a sensitivity of 100% a specificity of 92% and allows classifying correctly 93.94% of the patients, i.e., all but two patients with TRG2 who are incorrectly classified as complete responders. Using a cut point of .8905 gives a sensitivity of 37.50% a specificity of 100.00% and allows classifying correctly 84.85% of the patients (5 of the 8 complete responders are incorrectly classified as not complete responders, all the remaining 28 patients are correctly classified).

**Fig 6:**
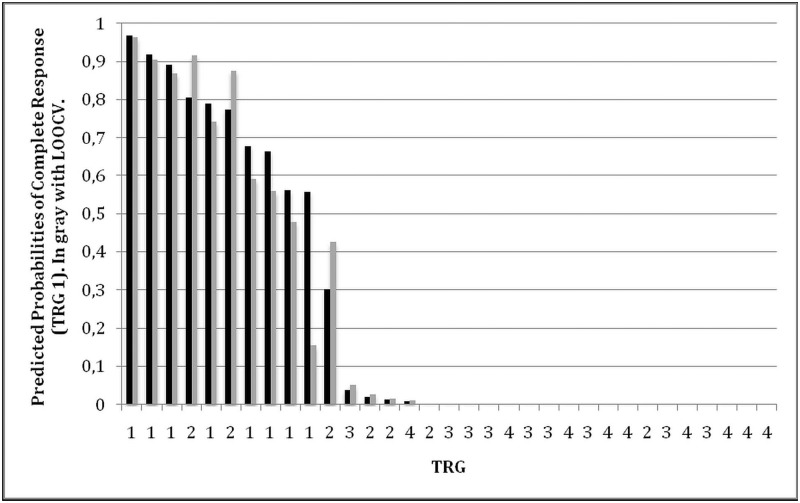
Prediction of complete response. In sample (black bars, TRG1) and out of sample (LOOCV, gray bars, TRG1).

Results for in-sample (black bars) and out-of-sample (gray bars) prediction accuracy of complete response for each individual patient are plotted in Fig 6.

The comparison of predicted probabilities with predicted probabilities with LOOCV reveals that the predictions of our model are robust to the exclusion of the patient’s observation from the training set.

## Discussion

Two previous studies [20, 21] have analyzed the responses to SCRT in LARC and have documented no significant metabolic responses to SCRT. One study [20] finds no correlation between metabolic response and TRG. On the basis of these results, it is generally accepted that in LARC measurements of metabolic response cannot be used to drive therapeutic strategies after SCRT.

Our results indicate that this conclusion is unwarranted. To understand why, it is important to indicate the reasons for which our analysis differs from the referred works.

The first reason is that, since we adopted a protocol with delayed surgery, we perform three PET scans, rather than two, and measurements of both metabolic and pathological response to SCRT are taken at later times. The first PET scan after SCRT (early PET) is performed on average 10.1 days after the end of SCRT, rather than the last day of radiotherapy or 2 days after; the second PET scan after SCRT (delayed PET) is performed on average 53 days after the end of SCRT; moreover, because surgery is performed on average 60.3 days after the end of SCRT, rather than 1 week after, TRG measurements provide an indication of the cumulative effect of SCRT after a longer period. This has three advantages. First, early PET measurements may be a more precise measurement of metabolic response; second, we have available an additional measure of metabolic response (delayed PET); third, TRG may provide a more reliable measurement of response when the effects of killing and apoptosis of tumor cells had probably had sufficient time to be fully detected on histopathological analysis [29, 30].

The second reason is that, rather than using the reduction in a single PET measurement, we consider the possibility of aggregating several measurements of metabolic response to make inferences about the likely pathological response.

The third reason is that, rather than simply asking what threshold gives the best classification of pathological responses, we provide a direct estimate of the probability of response for each individual patient.

Our analysis indicates significant metabolic responses to SCRT (SUVmax, SUVmean, or TLG) already at the early PET, as well as a clear correlation between these measurements of metabolic response and TRG. As previously mentioned, this may be due to the fact that we rely on more precise measurements of metabolic and pathological response. Despite being significant, the ability of these measurements to classify patients in terms of their TRG could not be considered satisfactory. Reductions in SUVmean and TLG classify correctly no more than 25 of 33 patients (or 75.76% of the total) as being major responders (TRG1-2) or nonresponders (TRG3-4).

A possible reason why reductions from baseline to early measurements perform poorly in classifying patients is that the metabolic response to SCRT may be slow. Our findings provide a moderate support to this view, given that reductions from baseline to delayed measurements in SUVmean and TLG allow a minor increase in the ability to classify patients correctly: reductions from baseline to delayed measurements in SUVmean and TLG make it possible to classify correctly 26 and 27 patients, respectively (or 78.79% and 81.82% of the total) as being major responders (TRG1-2) or nonresponders (TRG3-4). Even so, the ability to classify patients’ pathological responses to SCRT seems equally insufficient to propose clinically useful classification.

When we consider the possibility of aggregating different measures of metabolic behavior at different times with a logistic regression model, we find that the best specification is based on the level of TLG at the early PET and on the overall proportional reduction of TLG from the baseline to the delayed PET. The fact that TLG measurements are the best predictors of pathological response to SCRT is not entirely surprising. Previous statistical analyses of large samples of rectal cancer treated with CHRT [31] established a relation between tumor dimension and pathological response. TLG, as the product of SUV and metabolic volume, probably conveys information on both tumor dimension and metabolic behavior.

With our specification, based on TLG measurements, we obtain two results. The first is that we can classify correctly 32/33 patients (96.97%) as major responders (TRG1-2) or nonresponders (TRG3-4) and 31/33 patients (93.94%) as complete responders (TRG1) or not complete responders (TRG2-4). The second, more important, is that we provide simple formulas to compute the likelihood of an individual patient being a major responder or a complete responder on the basis of their TLG measurements. These simple formulas make it possible to estimate the probability of response prior to surgery. A more extensive patient panel would probably strengthen the prediction power, but there is evidence that the proposed formulas already provide references that should not be ignored for clinical purposes.

## Conclusion

Our findings show that logistic regression can be used to aggregate multiple metabolic measurements and achieve remarkable predictive ability of pathological response to SCRT. The straightforward prediction formulas we propose make it possible to assess the risks associated to LARC borne by a patient after SCRT. These risk assessments can be balanced against other health risks associated with further treatments and can therefore be used to make informed therapy adjustments after SCRT, for example, proceeding with complete surgery, practicing local excision, or leaning for a wait and see policy. Future research applying the same methodology will be useful to validate and fine-tune our formulas and ultimately improve the ability to predict pathological response.

## Supporting Information

S1 File Patient data. (XLSX)
